# A proposal to evaluate the management of tuberculosis programs: a qualitative, evaluability assessment in the border region of Brazil and Venezuela

**DOI:** 10.1590/0102-311XEN104823

**Published:** 2024-04-22

**Authors:** Débora de Almeida Soares, Ricardo Alexandre Arcêncio, Inês Fronteira

**Affiliations:** 1 Instituto de Higiene e Medicina Tropical, Universidade NOVA de Lisboa, Lisboa, Portugal.; 2 Escola de Enfermagem de Ribeirão Preto, Universidade de São Paulo, Ribeirão Preto, Brasil.; 3 Escola Nacional de Saúde Pública, Universidade NOVA de Lisboa, Lisboa, Portugal.

**Keywords:** Tuberculosis, Transients and Migrants, Border Health, Health Management, Tuberculose, Migrantes, Saúde na Fronteira, Gestão em Saúde, Tuberculosis, Migrantes, Salud Fronteriza, Gestión en Salud

## Abstract

This study aims to analyze the feasibility of building an evaluative model for the management of the Tuberculosis Prevention and Control Program in the State of Roraima, located on the border between Brazil and Venezuela. This is an evaluability assessment, a type of study used as a pre-evaluation of the development and implementation stages of a program, as well as throughout its execution. The study was developed in stages comprising the: (i) definition of the intervention to be analyzed and its objectives and goals; (ii) construction of the intervention logical model; (iii) screening of parties interested in the evaluation; (iv) definition of the evaluative questions; and (v) design of the evaluation matrix. Four priority components were defined for the evaluation: management of the organization and implementation of tuberculosis (TB) prevention and control policy; epidemiological surveillance management; care network management; and management of expected/achieved results. In this model, and based on theoretical references, we defined the necessary resources, activities, outputs, outcomes, and the expected impact for each of the policy management components. The management of the TB control program is feasible for evaluation based on the design of its components, the definition of structure and process indicators, and relevant results for the analysis of the management of TB prevention and control actions, as well as its influence on compliance with the agreed indicators and targets aiming at eradicating the disease by 2035.

## Abstract (pt)

Objetivou-se examinar a viabilidade da construção de um modelo avaliativo para a gestão do Programa de Prevenção e Controle da Tuberculose no Estado de Roraima, localizado na fronteira entre Brasil e Venezuela. Trata-se de um estudo de avaliabilidade, um tipo de estudo utilizado como pré-avaliação na fase de desenvolvimento e implementação de um programa, bem como ao longo de sua execução. O estudo foi desenvolvido em etapas: (i) definição da intervenção a ser analisada e seus objetivos e metas; (ii) construção do modelo lógico da intervenção; (iii) mapeamento dos interessados na avaliação; (iv) definição das questões avaliativas e (v) delineamento da matriz de avaliação. Foram definidos quatro componentes prioritários para avaliação: gestão da organização e implementação da política de prevenção e controle da tuberculose (TB), gestão da vigilância epidemiológica, gestão da rede de atenção à saúde e gestão dos resultados esperados/obtidos. Nesse modelo, e com base em referenciais teóricos, definimos os recursos, atividades, produtos, resultados e o impacto esperado para cada um dos componentes de gestão de políticas. A gestão do programa de controle da TB é passível de avaliação a partir do delineamento de seus componentes e da definição de indicadores de estrutura e processo, assim como de resultados relevantes e sua influência para o cumprimento das metas pactuadas, visando erradicar a doença até 2035.

## Abstract (es)

El objetivo fue analizar la viabilidad de elaborar un modelo de evaluación para la gestión del Programa de Prevención y Control de la Tuberculosis en el Estado de Roraima, que está en la frontera entre Brasil y Venezuela. Se trata de un estudio de evaluabilidad, un modelo de estudio que se utiliza como evaluación previa en la fase de desarrollo e implementación de un programa, así como a lo largo de su ejecución. El desarrollo del estudio se realizó en etapas: (i) definir la intervención a analizar y sus objetivos y metas; (ii) construir el modelo lógico de la intervención; (iii) mapear los interesados en la evaluación; (iv) definir las preguntas de la evaluación; y (v) trazar la matriz de evaluación. Se definieron cuatro componentes prioritarios para la evaluación: la gestión de la organización e implementación de la política de prevención y control de la tuberculosis (TB), la gestión de la vigilancia epidemiológica, la gestión de la red de atención a la salud y la gestión de los resultados esperados/obtenidos. En este modelo, y basándonos en referentes teóricos, definimos los recursos, actividades, productos, resultados y el impacto esperado para cada uno de los componentes de la gestión de políticas. La gestión del programa de control de la TB puede evaluarse a partir del diseño de sus componentes y de la definición de indicadores de estructura y proceso, así como de resultados relevantes y su influencia para el cumplimiento de las metas pactadas, con el fin de erradicar la enfermedad para 2035.

## Introduction

Tuberculosis (TB) is a disease that persists as a serious global public health problem, responsible for about 10 million people falling ill each year ^1^. Despite the existence of several viable protocols for TB control, a number of factors, such as limitations in the quality and effectiveness of programmatic actions developed by health systems for the prevention and control of this disease contribute to its persistence as an important condition, especially in developing countries ^2^.

Brazil is ranked among the 22 countries with the highest burden of TB, with around 80,000 new cases per year and 5,000 deaths ^3^. Among the Brazilian states with the highest burden of the disease are Amazonas (71.3/100,000 inhabitants), Rio de Janeiro (67.4/100,000 inhabitants), Roraima (54.6/100,000 inhabitants), Acre (50.3/100,000 inhabitants), and Pernambuco (45.9/100,000 inhabitants) ^4^.

In Brazil, from 2015 to 2021, the total number of TB cases in vulnerable populations, including migrants, increased significantly. From 2015 to 2019, outbreaks of measles, hepatitis A, TB, malaria, syphilis, and leishmaniasis in groups of Venezuelan immigrants living on the border between Brazil and Venezuela, Roraima State ^5^, were reported to the Information System for Notifiable Diseases (SINAN, acronym in Portuguese) of the Brazilian Ministry of Health. All the diseases reported in immigrants showed higher numbers than those recorded in the national population living in that territory.

Since 2015, the State of Roraima has been dealing with the intense migration of Venezuelans motivated by the country’s political, economic, and social crisis, which began in 2013 ^6^. Even before the raise in immigration, the State of Roraima already showed significant numbers of TB in its resident population. According to the Epidemiological Bulletin of the Brazilian Ministry of Health, in 2014 Roraima recorded an incidence rate of 29.7 cases/100,000 inhabitants, ranking in the northern Brazilian states with the highest TB incidence ^7^.

A total of 2,111 cases of TB were reported in Roraima from 2009 to 2019 in SINAN. Of these, a total of 49 (2.4%) notified cases of TN were individuals from the State of Amazonas, which borders the national territory with the State of Roraima and 18 (10.9%) new cases were reported in immigrants from other countries, most from Venezuela 132 (72.9%) ^8^. In 1999, the Brazilian Ministry of Health created the Brazilian National Tuberculosis Control Program (PNCT, acronym in Portuguese), with the objective of expanding TB control actions in the country and reducing the prevalence of the disease in the population ^9^. In 2014, the World Health Organization (WHO) approved a new global strategy to fight TB, with the strategic goal of eliminating the disease by 2035 ^10^. However, achieving these goals depends on a strategic planning of actions based on a situational diagnosis of local needs, with the incorporation of monitoring routines and evaluation of the results achieved ^11^.

Even amid the constant implementation of national, state, and municipal health policies and programs, the Brazilian Unified National Health System (SUS, acronym in Portuguese) is facing a global crisis regarding the effective organization and management of health services and actions, increasingly unable to meet the real and specific demands of each health territory, especially in remote regions such as border regions ^11^ ^,^ ^12^ ^,^ ^13^.

Despite the existence of an international health regulation that guides appropriate surveillance practices at international borders, the guidelines prioritize large-scale events caused by rapidly spreading infectious diseases ^14^ and this, depending on the priorities of management action, brings the risk of neglecting TB, especially its resistant forms, which should require priority surveillance.

Previous studies have widely proposed evaluating the implementation of health programs, actions, and services ^2^ ^,^ ^6^ ^,^ ^9^. However, the evaluation of the management of these programs is still incipient and remains a challenge in the field of evaluative research.

Considering the epidemiological profile of TB in the State of Roraima and, more specifically, on the border between Brazil and Venezuela, we identified the need to evaluate the management process of the Tuberculosis Prevention and Control Program in this state, in order to analyze the degree of implementation of policy management and the degree of management influence on the implementation of TB prevention and control actions.

This study aims to examine the feasibility of building an evaluative model for the management of the Tuberculosis Prevention and Control Program in the State of Roraima.

## Material and methods

This is an evaluability assessment study. Evaluability assessment is a type of study used as a pre-evaluation of the development and implementation stages of a program, as well as throughout its execution ^15^. The study was developed in five stages: (i) definition of the intervention to be analyzed and its objectives and goals; (ii) construction of the logical model of the intervention; (iii) screening of parties interested in the evaluation; (iv) definition of the evaluative questions; and (v) design of the evaluation matrix.

We identified the Tuberculosis Prevention and Control Program, its objectives and goals as the target of our evaluation, especially regarding management processes.

The logical model allows us to visualize, in a systematic and detailed way, the components of an intervention to be analyzed ^16^. By constructing the logical model, it is possible to specifically describe the elements that make up the structure, process, and expected results of the intervention ^15^.

**Figure 1:**
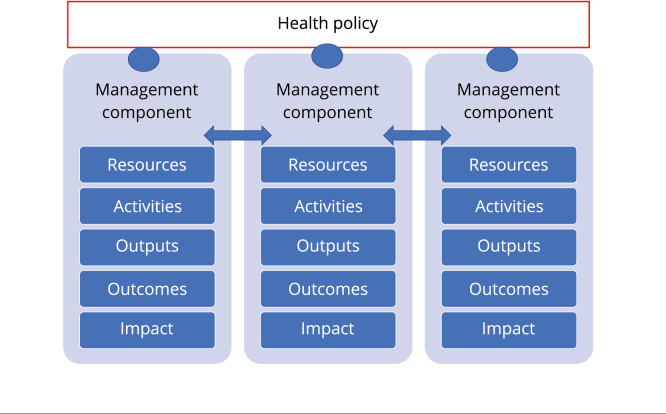
Schematic representation of the logical evaluation model of a health policy.

Source: prepared by the authors.

To build the logical model of management evaluation, the manuals and technical guidelines recommended by the Brazilian Ministry of Health ^10^ ^,^ ^17^ ^,^ ^18^ ^,^ ^19^ for implementing TB prevention and control strategies in the territories where they operate were used as theoretical references. In this model, and based on the theoretical references, we defined the necessary resources, activities, outputs, outcomes, and expected impact for each of the policy management components, according to the scheme in Figure 1.

After this phase, the logical model was analyzed by five specialists in health evaluation and planning who independently collaborated to adjust and validate the construct. These specialists included three doctoral professors specialized in health evaluation and two health policy managers interested in the evaluation: one from the health evaluation area and the other from the health surveillance area. Three specialists were contacted via emailing and the other two via face-to-face, individual meetings. At contact, the objectives of the research and the matrix to be validated were explained. The experts only had access to the matrix after agreeing to take part in the research. Anonymity and confidentiality of the raters’ identity were preserved.

Four specialists were randomly selected to analyze all the components of the matrix and suggest any necessary adjustments. The acceptability of each item in the matrix components was assessed using a form containing the following answer alternatives: agree, partially agree and suggest adjustments, and disagree. For each item evaluated, a total percentage > 65% agreement between the specialists was considered. In case of a tie between the answers, the 5th judge was responsible for analyzing the questions again and deciding with greater weight on the permanence, adjustment, or exclusion of the item evaluated. The evaluation of the experts was carried out over one month. After the evaluation, the final product was applied in a pilot test in two priority municipalities for managing TB policy in the State of Roraima.

A construct validation of the logical model and matrices was carried out, especially regarding the clarity of the content and the relation of the items to be evaluated with the objectives of the analysis. Once the logical model had been constructed, those interested in the evaluation were listed and the evaluative questions were formulated, especially considering the relevance and feasibility of the information that could be obtained with each question and the relation with the logical model of the intervention to be evaluated.

The matrix of evaluative questions was elaborated based on the theoretical framework used to construct the logical model of the program. Based on the evaluative questions, the evaluation design was built, where the items to be evaluated, the evaluation parameters, and the instruments to be used for each step of the process were categorized (Box 1). Other six matrices already validated in previous studies were also considered and adapted for this study ^2^ ^,^ ^6^ ^,^ ^20^ ^,^ ^21^ ^,^ ^22^ ^,^ ^23^.

**Box 1:** Judgment matrix.

COMPONENTS	PRIORITY OBJECTIVES FOR ANALYSIS/CALCULATION	CRITERIA OF ANALYSIS	SOURCE OF ANALYSIS
Management of the organization and implementation of TB provention and control policy			
1. Management time	The fact that there is a reasonable minimum period for recognizing the policy and the attributions inherent in planning and implementing actions in the territory	More than 6 months and less than 1 year	Interview
		1 year or more	
2. Recognizes the epidemiological profile of TB in the territory of operation	Recognition of epidemiological, social, and clinical aspects, outcomes, and indicators related to reported TB cases	Does not recognize	Interview
		Partially recognizes	
		Fully recognizes	
3. Preparation of annual action planning for TB control	Existence of a periodic work plan, based on actions, goals, and financial incentives	There is no work plan	Interview and official documents: work plans, activity reports
		There is a work plan	
4. Planning actions in accordance with the technical-operational guidelines and regulations established for PNCT in Brazil	Use of guidance manuals and technical notes issued by the Brazilian Ministry of Health to draw up work plans, planning of goals, activities, and indicators to be implemented in the territories	There is no work plan	Interview and official documents: work plans, activity reports
		There is a work plan, but it lacks coherence and correlation with the guidelines and regulations	
		There is a work plan prepared that is coherent and correlates with the PNCT guidelines and regulations	
5. Implementation of intersectorial planning of actions integrated with technical areas of related policies for the prevention and control of TB	Workplan presents goals and joint actions with other areas and/or coordination of health policies (HIV/AIDS, women’s health, child health, men’s health, Indigenous health, among others)	There is no work plan	Interview and official documents: work plans, activity reports
		There is a work plan, but it does not show the planning of goals and intersectorial actions	
		Work plan presents the planning of goals and actions of other related policy coordination in a timely and fragmented way	
		Work plan presents comprehensive integrated planning of goals and intersectorial actions	
6. Integrated planning of actions with the government and other institutions of civil society for actions to be carried out for vulnerable populations at risk of TB, including international migrants	Work plan presenting actions and goals established with other federal, state, and municipal institutions, as well as nongovernmental institutions that work in the context of vulnerable populations (including international migrants), namely the government, unified system of social assistance, associations, and social organizations, among others	There is no work plan	Interview and official documents: work plans, activity reports
		Work plan does not present planning of goals and actions established with other federal, state, and municipal institutions, as well as nongovernmental institutions that act directly with vulnerable populations	
		Work plan presents the planning of goals and actions established with other federal, state, and municipal institutions, as well as nongovernmental institutions that act directly with vulnerable populations	
		Work plan presents comprehensive integrated planning of goals and actions established with other federal, state, and municipal institutions, as well as nongovernmental institutions that act directly with vulnerable populations	
7. Planning and execution of financial budget for the provision of materials necessary for TB control actions	Planned activities with defined financial budget to be carried out during the term of the work plan	There is no defined budget plan	Interview and official documents: work plans, activity reports
		There is a defined budget plan, but there is no financial execution according to budget forecast	
		There is financial execution in accordance with the budget forecast	
Management of epidemiological surveillance			
1. Provides SINAN for reporting and TB cases	SINAN is implemented in all health units that provide care to patients with suspected TB, at all levels of care: primary, medium, and high complexity	SINAN not implemented	Interview
		SINAN implemented but not available	
		SINAN implemented and available	
2. Recognizes, makes available, and supervises other information systems related to TB case reporting	Information systems implemented by the Brazilian Ministry of Health where special situations related to TB are reported at all levels of care (individualized therapeutic regimens, resistant TB, pregnant women)	Reporting systems for special cases of TB not implemented	Interview
		Systems for reporting special cases of TB implemented but not available	
		Special TB case notification systems implemented and available	
3. Periodically monitors and analyzes health indicators related to TB control	Recognition and use of the main indicators related to TB prevention and control as a planning and monitoring tool, and analyzes them with bulletins and historical series	Does not recognize the indicators and does not use them as a management tool	Interview
		Recognizes the indicators but does not use them as a management tool	
		Recognizes, uses, and analyzes indicators as a management tool	
4. Periodically carries out the quality analysis of the data entered in SINAN	Periodic reviews the SINAN database to analyze and correct possible inconsistencies related to the notification and monitoring of TB cases until the case is closed	Does not periodically review the database	Interview and official documents: work plans, activity reports
		Reviews database punctually and in fragments, not covering all the cases in the system	
		Reviews database periodically, covering all the cases in the system, until closure	
5. Manages and/or monitors cases in special treatments for TB	Mapping and monitoring of cases of drug-resistant TB in children, pregnant women, and people with comorbidity, among other special situations	Does not map and/or monitor cases in special treatment for TB	Interview
		Maps and monitors cases in special treatment for TB	
6. Recognizes territories and special populations most vulnerable and at risk of TB disease, including international migrants	Identification and mapping of areas with higher incidence of the disease in the general population and in special populations, considered to be more vulnerable, including international migrants	Does not map areas with higher incidence of TB in the general population and in special populations or areas at greater risk and vulnerability to TB	Interview and official documents: work plans, activity reports
		Maps areas with higher incidence of TB only for the general population, without considering special populations or areas at greater risk and vulnerability to TB	
		Maps areas with higher incidence of TB in the general population and in special populations or areas at greater risk and vulnerability to TB	
7. Maps and prioritizes areas with the highest incidence of cases for TB control actions	Priority in planning and implementing actions in territories and areas with the highest number of new cases reported and under follow-up for clinical TB treatment	There is no priority in planning and implementing actions in territories and areas with a higher number of new cases reported and under follow-up for clinical treatment of TB	Interview and official documents: work plans, activity reports
		There is a priority plan for implementing actions in territories and areas with the highest number of new cases reported and under follow-up for clinical treatment of TB	
8. Articulates, plans, and/or carries out cross-border TB surveillance actions	Elaboration and/or intersectorial and inter-institutional plan of actions and goals that include epidemiological surveillance for TB prevention and control at the international and national borders of the territory	There is no action plan for the prevention and control of TB at borders	Interview and official documents: work plans, activity reports
		There is an action plan prepared for the prevention and control of TB at borders, but it is not implemented	
		There is an action plan prepared for the prevention and control of TB at borders, and it is fully implemented	
9. Periodically issues epidemiological bulletins related to TB	Preparation and dissemination of epidemiological bulletins that present data on new cases, outbreaks, epidemics, and results of health indicators related to TB in the territories	Does not prepare bulletins	Interview and official documents: work plans, activity reports
		Prepares newsletters, but does not disclose them	
		Prepares bulletins, but discloses them in a punctual and restricted way, not covering access to all professionals involved in TB prevention and control	
		Prepares epidemiological bulletins regularly and widely	
10. Periodically issues management reports on the actions taken to control TB	Preparation and dissemination of reports on the activities carried out, goals achieved, and results of indicators established for the prevention and control of TB in the territories	Does not prepare reports	Interview and official documents: work plans, activity reports
		Prepares specific reports or only on request, but does not disclose activities carried out, goals achieved, and results of indicators established for the prevention and control of TB in the territories	
		Prepares and disseminates reports on the activities carried out, goals achieved, and results of indicators established for the prevention and control of TB in the territories	
Management of the care network			
1. Promotes and supervises the notification of all confirmed cases of TB	Periodic supervision to verify the implementation of TB cases notification in all health units at all levels of care in the territories of operation	Does not carry out supervision	Interview and official documents: work plans, activity reports
		Carries out punctual supervision or only on request to verify the implementation of TB case notification in all health units at all levels of care, in the territories of operation	
		Periodically carries out supervision to verify the implementation of TB case notification in all health units at all levels of care, in the territories of operation	
2. Articulation with the reference laboratory network to provide TB diagnostic tests	Preparation and execution of a joint action plan with the laboratory network to ensure the provision of diagnostic imaging tests and other diagnostic tests that are recommended by the PNCT technical regulations (sputum bacilloscopy, culture, X-ray, RMT, sensitivity test, HIV testing, TST, BCG vaccination)	There is no action plan and no laboratory network offering laboratory tests for TB diagnosis	Interview and official documents: work plans, activity reports
		There is a plan of joint actions implemented and laboratory network offering tests for TB diagnosis	
3. Coordination and supervision of the active search for respiratory symptoms	Supervision and implementation of actions to raise awareness among health professionals for active search for respiratory symptoms in the territories	Not carried out	Interview
		Supervises and implements actions to raise awareness among health professionals about active search for respiratory symptoms in the territories, only on time or on demand	
		Periodically supervises and implements actions to raise awareness among health professionals about active search for respiratory symptoms in the territories	
4. Implementation of improvement and qualification actions on TB for professionals who work directly in the diagnosis and treatment of cases (FHS, outpatient clinics, hospitals)	Promotion and implementation of qualification, updating, and/or professional improvement actions for all health professionals working in the clinical management of TB, at all levels of assistance	Does not promote or carry out qualification, updating, and/or professional improvement actions for all health professionals who work in the clinical management of TB, at all levels of assistance	Interview
		Promotes or carries out qualification, updating, and/or professional improvement actions in a punctual and fragmented way, not covering all health professionals who work in the clinical management of TB, at all levels of assistance	
		Promotes or carries out qualification, updating, and/or professional improvement actions periodically and for all health professionals who work in the clinical management of TB, at all levels of assistance	
5. Encourages and fosters educational practices for the community on TB prevention and control	Development and/or distribution of educational materials on TB prevention and control for health units at all levels of care	Does not prepare or provide educational materials	Interview and official documents: work plans, activity reports
		Prepares and/or provides educational materials on TB prevention and control in a timely and fragmented way	
		Prepares and/or provides educational materials on TB prevention and control for health units of all levels of care	
6. Implementation and monitoring of DOT at all levels of care	Elaborates an action plan and supervises the implementation of DOT in the territories	There is no DOT implemented	Interview and official documents: work plans, activity reports
		There is an action plan designed to implement the DOT, but it is executed in a timely and fragmented manner	
		There is an action plan prepared for implementing the DOT and it is fully executed and periodically supervised	
7. Performs provision and logistical control of medicines and supplies necessary for the clinical management of TB in the territory of operation	Planning, supervision, and control of the distribution of medicines and inputs used in the clinical management of TB with the logistics and distribution centers and pharmacies of health units at all levels of care	Planning, supervision, and control of the distribution of medicines used in the clinical management of TB with logistics and distribution centers and pharmacies of health units at all levels of care	Interview and official documents: work plans, activity reports
		Planning, supervision, and control of the distribution of medicines used in the clinical management of TB with the logistics and distribution centers and pharmacies of health units at all levels of care	
8. Supervising and participating in the planning of bacteriological diagnosis and quality control actions at the reference laboratory	Joint elaboration of measures for the implementation and supervision of quality control of samples and laboratory analyses carried out in the diagnostic reference laboratories for TB in the territories	Does not supervise and participate in the planning of bacteriological diagnosis and quality control actions with the reference laboratory	Interview
		Supervises and participates in the planning of bacteriological diagnosis and quality control actions with the reference laboratory in a timely manner, on demand	
		Periodically supervises and participates in the planning of bacteriological diagnosis and quality control actions with the reference laboratory	
9. Implementation of a specific action plan for special populations or populations at greater risk and vulnerability for TB disease in each territory, including international migrants	Elaboration and implementation of work plans that include specific health activities, goals, and indicators for special populations or those at greater risk and vulnerability for TB, based on the epidemiological profile of TB in the territories where it operates (including international immigrants), seeking to expand and facilitate access for these groups to the means of prevention, diagnosis, and treatment of TB	There is no specific action plan for special populations or populations at greater risk and vulnerability to TB in each territory	Interview and official documents: work plans, activity reports
		There is a specific action plan for special populations or populations at greater risk and vulnerability to TB in each territory, but it is not implemented	
		There is a specific action plan for special populations or populations with higher risk and vulnerability to TB in each territory, but it is executed on time	
		There is a specific action plan for special populations or populations with greater risk and vulnerability to TB in each territory, and it is implemented in an integral manner	
10. Implementation of latent TB diagnosis in health units at all levels of care	Provision of inputs and training for health professionals for the diagnosis and treatment of latent TB	There is no implementation of actions for the diagnosis of latent TB in the territory	Interview and official documents: work plans, activity reports
		The implementation of actions for the diagnosis of latent TB is carried out in a punctual and fragmented way	
		The implementation of actions for the diagnosis of latent TB is carried out continuously and integrally	
Management of expected/obtained results			
1. Compliance with the goals established in the annual planning	Preparation and periodic issuance of a management report containing results related to goals, indicators, and planned activities	There was no compliance with established goals	Interview and data from official documents
		Proportion of achievement of established goals was less than 60% of planned goals	
		Proportion of achievement of established goals was equal to or greater than 60% of the planned goals	
2. Execution of planned activities per quarter	Periodic issuance of information on activities carried out for the prevention and control of TB in the territories	Did not carry out the planned activities	Interview and data from official documents
		Partially carried out the planned activities	
		Carried out all planned activities	
3. Incidence coefficient of TB in the territory	Estimation of the risk of TB (number of new TB cases, divided by population, multiplied by 100,000)	10 cases per 100,000 inhabitants	Data from SINAN and management reports and bulletins
		Equal to 10 cases per 100,000 inhabitants	
		Less than 10 cases per 100,000 inhabitants	
4. TB mortality rate in the territory	Estimating the risk of death from TB (number of deaths with underlying cause TB, divided by population, multiplied by 100,000)	There was a reduction in the TB mortality rate	Data from SINAN and management reports and bulletins
		There was a maintenance of the TB mortality coefficient	
		There was an increase in the TB mortality rate	
5. Proportion cured after 6 months of treatment	Measuring the success of TB treatment and the consequent decrease in disease transmission (total of new cases of pulmonary TB terminated with diagnosis of cure x 100/total of new cases of TB diagnosed)	Less than 75 % of TB cases cured	Data from SINAN and management reports and bulletins
		At least 75% of TB cases cured	
		At least 85% of TB cases cured	
		More than 85% of TB cases cured	
6. Proportion of treatment abandonment	Measurement of the proportion of patients who abandon treatment, remaining absent for more than 60 days after the last visit or remaining without medication for more than 30 days (total of TB cases terminated by treatment abandonment x 100/total of TB cases reported)	Less than 5% of cases	Data from SINAN and management reports and bulletins
		Equal to 5% of cases	
More than 5% of cases

DOT: directly observed treatment; FHS: Family Health Strategy; PNCT: Brazilian National Tuberculosis Control Program; RMT: rapid molecular test; SINAN: Brazilian Information System for Notificable Diseases; TB: tuberculosis; TST: tuberculin skin test.

## Results

The theoretical framework enabled the identification of the competences established for the management of the TB control program at the different administrative levels (i.e., state and municipal), as well as the criteria that should be considered for each strategy without implementation. To construct the logical model, we considered the guidelines of pillars 1 (prevention and integrated people-centered care) and 2 (bold policies and support system) of the Brazilian National Plan to End Tuberculosis as a Public Health Problem (2021-2025) and the attributions of each federal entity to comply with the plan ^10^.

Four priority components were defined for evaluation: management of the organization and implementation of TB prevention and control policy, management of epidemiological surveillance, management of the care network, and management of the expected/obtained results. For each component, strategic activities considered essential for the effective management of the program were added and activities, products, results, and expected impact in terms of TB-related health indicators were defined. The evaluation components developed are directly related to the structure, process, and results used in health policy evaluation (Box 2).

**Box 2:** Logical evaluation model.

	COMPONENTS			
	MANAGEMENT OF THE ORGANIZATION AND IMPLEMENTATION OF TB PREVENTION AND CONTROL POLICY	MANAGEMENT OF EPIDEMIOLOGICAL SURVEILLANCE	MANAGEMENT OF THE CARE NETWORK	MANAGEMENT OF EXPECTED/OBTAINED RESULTS
Resources	Budgetary planning	Financial resources	Financial resources for logistics and development	Work infrastructure
	Work plan	Work plan	Work plan	Work plan
	Intra- and intersectorial joints	Indicators and goals	Indicators and goals	Indicators and goals
	Indicators and goals	Human resources	Human resources	Human resources
	Human resources			
Activities	1. Recognition and mapping of the epidemiological profile of TB	1. Provides SINAN for reporting and TB cases	1. Promotes and supervises the notification of all confirmed TB cases	1. Compliance with the goals established in the annual planning
	2. Drafting of a work plan	2. Acknowledges, makes available, and supervises other information systems related to TB case reporting		2. Implementation of activities planned on a quarterly basis
	3. Planning actions in accordance with the technical-operational guidelines and regulations established for the PNCT	3. Periodic monitoring and analysis of health indicators related to TB control	2. Articulation with the reference laboratory network for the provision of TB diagnostic tests	3. TB incidence in the territory
	4. Carrying out intra-sectoral planning of integrated actions with the technical areas of related policies for TB prevention and control	4. Carries out periodic quality analysis of data entered in SINAN		4. TB mortality rate in the territory
	5. Integrated planning of actions with the government and other institutions of civil society for actions to be carried out with populations at greater risk of TB, including international migrants	5. Manages and/or monitors cases in special treatments for TB	3. Coordination and supervision of the active search for respiratory symptoms	5. Proportion cured after 6 months of treatment
	6. Planning and implementation of the financial budget for the provision of materials necessary for TB control actions	6. Recognizes territories and special populations most vulnerable and at risk of TB, including international migrants		6. Proportion of treatment dropout
		7. Maps and prioritizes areas with higher incidence of cases for TB control actions	4. Implementation of improvement and qualification actions on TB for professionals who work directly in the diagnosis and treatment of cases (FHS, outpatient clinics, hospitals)	
		8. Articulates, plans and/or carries out cross-border TB surveillance actions		
		9. Periodically issues epidemiological reports related to TB	5. Encourages and fosters educational practices for the community on TB prevention and control	
		10. Periodically issues management reports on actions taken to control TB	6. Implementation and monitoring of DOT at all levels of care	
			7. Provision and logistical control of medicines and supplies necessary for the clinical management of TB in the territory of operation	
Outputs	1. Recognition of epidemiological, social, and clinical aspects, outcomes, and indicators related to reported TB cases	1. SINAN implemented and available	1. Supervision of notification of confirmed TB cases carried out	1. Established goals met/ achieved
	2. Existence of a periodic work plan, based on actions, targets, and financial incentives	2. Special TB case reporting systems implemented and available	2. Plan of joint actions implemented and laboratory network offering tests for TB diagnosis	
	3. Elaborated work plan that is coherent and correlates with the PNCT guidelines and regulations	3. Use of key indicators related to TB prevention and control	3. Supervision and implementation of actions to raise awareness among health professionals about the active search of respiratory symptoms in the territories	2. Planned activities carried out
		4. Periodic review of the SINAN database	4. Improvement and qualification actions on TB implemented	
	4. Work plan featuring integrated planning of goals and intersectorial actions	5. Maps and monitors cases in special treatment for TB	5. Prepares and/or provides educational materials on TB prevention and control for health units at all levels of care	3. Less than 10 cases per 100,000 inhabitants
		6. Areas with the highest incidence of the disease in the general population and in special populations considered most vulnerable, including international migrants, mapped	6. Action plan prepared for the implementation of the DOT and implemented and supervised	
	5. Work plan presenting integrated planning of goals and actions established with other federal, state, and municipal institutions, as well as nongovernmental institutions that work directly with vulnerable populations, including international migrants	7. Priority plan of actions in the territories and areas with the highest number of new cases reported and being followed up for clinical treatment of TB implemented	7. Planning, supervision, and control of the distribution of medicines and inputs used in the clinical management of TB	4. Reduction in the TB mortality rate each year
	6. Financial execution of the work plan, in accordance with the budget forecast	8. Action plan for TB prevention and controlat borders implemented	8. Supervision and participation in the planning of bacteriological diagnosis and quality control actions with the reference laboratory carried out periodically	
		9. Epidemiological bulletins issued periodically	9. Specific action plan for special populations or populations at greater risk and vulnerability for TB in each territory implemented	5. A minimum of 85% of TB cases cured 6. Less than 5% of cases
		10. Management reports issued periodically	10. Actions to diagnose latent TB implemented	
Outcomes	Strategic planning and promotion of actions to ensure the implementation of TB prevention and control activities with adequate resources (human, infrastructure, and financial)	Implementation of TB prevention and control actions based on evidence and the needs of each territory	Intensification of TB prevention actions Early diagnosis of all forms of TB	Assertiveness and efficiency in the implementation of TB prevention and control
	Strengthening the role of health surveillance management based on the co-participation of public agents and civil society in TB prevention and control actions	Implementation of quality computerized case registration systems for more timely decision making	Adequate and timely treatment of all diagnosed cases of TB aiming for comprehensive care	Effectiveness of actions planned and implemented for the prevention and control of TB in the territories
	Strengthening intra- and intersectoral articulation to ensure the humanization of TB prevention and control actions	Implementation of new health care technologies for TB prevention and control based on the use of strategic information	Implementation of effective actions to ensure access to adequate diagnosis and treatment for population groups with greater vulnerability and risk of TB	Results of health indicators compatible with the recommended/established parameters
	Sustainability of the operational capacity of health management to act in TB prevention and control	Expansion of the resolution capacity of health surveillance management	Qualification and training of health professionals for the proper clinical management of TB	
		Implementation of TB epidemiological surveillance actions in border regions	Rational use of materials and medicines	
Impact	REDUCTION IN THE INCIDENCE OF TB			
	REDUCTION IN TB MORTALITY			
PROGRESSIVE ELIMINATION OF TB BY 2035			

DOT: directly observed treatment; FHS: Familiy Health Strategy; PNCT: Brazilian National Tuberculosis Control Program; SINAN: Brazilian Information System for Notificable Diseases; TB: tuberculosis.

Based on the construction of the logical model, four evaluative questions emerged and were defined to analyze the degree of implementation of the management of the program’s structure, process, and results: (1) What actions are taken and what work is carried out by the managing entity to control TB? (2) How are the actions planned and monitored? (3) What is the manager’s perception of the impact of migration on the epidemiological profile of TB? (4) What are the challenges faced in managing the TB prevention and control policy?

The following aspects were considered: management profile and qualification, management autonomy and financial support for decision-making, coherence and assertiveness among decision-making, program objectives, territory reality, capacity of articulation of the healthcare network, strategic planning culture, and quality of the results obtained in decision-making (Box 3). These analysis items triggered the construction of the judgment matrix and the 34 parameters to be analyzed (Box 1).

**Box 3:** Dimensions for analyzing the degree of implementation of the management of structure, process and results by component.

MANAGEMENT OF THE ORGANIZATION AND IMPLEMENTATION OF TB PREVENTION AND CONTROL POLICY	MANAGEMENT OF EPIDEMIOLOGICAL SURVEILLANCE	MANAGEMENT OF THE CARE NETWORK	MANAGEMENT OF EXPECTED/OBTAINED RESULTS
1. Management time	1. Provides SINAN for reporting and TB cases	1. Promotes and supervises the notification of all confirmed TB cases	1. Compliance with the goals established in the annual planning
2. Recognizes the epidemiological profile of TB in the territory of operation	2. Recognizes, makes available, and supervises other information systems related to TB case reporting	2. Articulation with the reference laboratory network for the provision of TB diagnostic tests	
3. Preparation of annual action planning for TB control	3. Periodic monitoring and analysis of health indicators related to TB control	3. Coordination and supervision of the active search for respiratory symptoms	2. Implementation of activities planned on a quarterly basis
4. Planning actions in accordance with the technical-operational guidelines and regulations established for PNCT in Brazil	4. Periodically carries out the quality analysis of the data entered in SINAN	4. Implementation of improvement and qualification actions on TB for professionals who work directly in the diagnosis and treatment of cases (FHS, outpatient clinics, hospitals)	
5. Implementation of intersectoral planning of integrated actions with technical areas of related policies for the prevention and control of TB	5. Manages and/or monitors cases in special treatments for TB	5. Encourages and fosters educational practices for the community on TB prevention and control	3. TB incidence in the territory
6. Integrated planning of actions with the government and other institutions of civil society for actions to be carried out for populations at risk of TB vulnerability, including international migrants	6. Recognizes territories and special populations most vulnerable and at risk of TB, including international migrants	6. Implementation and monitoring of DOT at all levels of care	
7. Planning and execution of financial budget for the provision of materials necessary for TB control actions	7. Maps and prioritizes areas with the highest incidence of cases for TB control actions	7. Provides and controls the logistics of medicines and supplies necessary for the clinical management of TB in the territory of operation	4. TB mortality rate in the territory
	8. Articulates, plans, and/or carries out cross-border TB surveillance actions	8. Supervision and participation in the action planning of bacteriological diagnosis and quality control at the reference laboratory	
	9. Periodically issues epidemiological bulletins related to TB	9. Implementation of a specific action plan for special populations or populations at greater risk and vulnerability to TB disease in each territory, including international migrants	5. Proportion cured after 6 months of treatment
10. Periodically issues management reports on the actions taken to control TB	10. Implementation of latent TB diagnosis in health units at all levels of care	6. Proportion of treatment dropout

DOT: directly observed treatment; FHS: Family Health Strategy; PNCT: Brazilian National Tuberculosis Control Program; SINAN: Brazilian Information System for Notificable Diseases; TB: tuberculosis.

## Discussion

This pre-evaluation study highlights the relevance of incorporating health evaluation activities into the context of political-institutional management of a health program so that the implementation of health actions can result in the achievement of the proposed goals and objectives, in this particular case, for TB.

The management of health services is an administrative practice that aims to optimize the functioning of organizations to obtain results that reflect efficiency in work relationships, effectiveness in achieving objectives and goals, and effectiveness in solving health demands ^24^. In the context of the SUS, results-oriented management should adopt evaluation as an activity integrated into public management and the functioning of the political system, using evaluative research as an instrument to support these practices ^25^.

Health evaluation should be used as a routine practice for strategic management, aiming at improving the actions and services offered to the population. However, several authors point to the incipient culture of health evaluation in Brazil, especially in the scope of evaluating the management methods implemented for these policies to be carried out ^2^ ^,^ ^24^ ^,^ ^26^.

The Brazilian Ministry of Health, in its technical guidelines, describes in detail the duties inherent in the management of the TB program, at all federal instances ^18^. In this sense, the management of the TB control policy, at all spheres of government, should be based on the theoretical and methodological foundations of health surveillance and the protocols produced for this purpose, and also on a prior evaluation that allows a broad view of the components of the operationalization of actions and identification of unmet needs for improvement and correction in the plan to be developed ^27^.

Based on these theoretical references, it was possible to select and describe which activities are essential in health management component so that the intervention can be effectively implemented in the territory. Previous studies related to the evaluation of TB control programs were used as a reference for the construction of the judgment matrix and the evaluation parameters of this study ^2^ ^,^ ^6^ ^,^ ^20^ ^,^ ^21^ ^,^ ^22^ ^,^ ^23^. Health management components were mainly analyzed and adapted to the evaluative objectives of this research.

The evaluation of actions and public policies in border regions is considered paramount for resolving the specific demands of this context ^6^. Considering that TB holds a significant impact on the epidemiological profile of Roraima, which neighbors Venezuela, evaluative items related to the strategic planning of TB control actions at the international borders of the territory were included in the judgment matrix.

The logical model and judgment matrix developed are instruments capable of consolidating the priorities for effective program management and can be considered valid evaluative models for application in various contexts, enabling strategic management and assertive decision-making ^21^. In this study, we consider that the priority analysis objectives addressed in each component presented an interdependent relationship capable of allowing the achievement of results and the expected impact of the actions carried out by program management.

Still, we identified some limitations to carry out a validity study. This, however, does not prevent the study from being implemented: the limitation of theoretical references available for Brazil and, more specifically, for Roraima and the dependence on the willingness of managers and other stakeholders to participate in such a study. In addition, because it is a validity study, the validation process was carried out with a more simplistic methodology in order to demonstrate whether the management of the TB program is evaluable.

## Final considerations and conclusions

Evaluation studies are fundamental for decision-making on the implementation of an intervention and can be used from the theoretical conception to the measurement of the results obtained. The management of the TB control program is feasible for evaluation based on the design of its components and the definition of structure, process indicators, and relevant results for the analysis of the management of TB prevention and control actions and its influence on the achievement of the agreed indicators and goals: the eradication of the disease by 2035.

This process of evaluating the management of the program in Roraima is relevant when considering the epidemiological profile of TB and the impact of international migration in this context, which demands a specific management approach to the peculiarities inherent to this public health problem in this territory.
